# Harmonizing technological advances in phenomics and genomics for enhanced salt tolerance in rice from a practical perspective

**DOI:** 10.1186/s12284-019-0347-1

**Published:** 2019-12-04

**Authors:** Sarika Jaiswal, R. K. Gautam, R. K. Singh, S. L. Krishnamurthy, S. Ali, K. Sakthivel, M. A. Iquebal, Anil Rai, Dinesh Kumar

**Affiliations:** 1 Centre for Agricultural Bioinformatics, ICAR-Indian Agricultural Statistical Research Institute, PUSA, New Delhi, 110012 India; 2 Division of Field Crop Improvement & Protection, ICAR-Central Island Agricultural Research Institute, Port Blair, Andaman and Nicobar Islands 744105 India; 3 Division of Plant Breeding Genetics and Biotechnology, International Rice Research Institute, DAPO Box 7777, Los Banos, Metro Manila Philippines; 4 Division of Crop Improvement, ICAR-Central Soil Salinity Research Institute, Karnal, Haryana 132001 India

**Keywords:** Genomics, Genome editing, Phenomics, QTL mapping, Rice molecular breeding, Salinity tolerance, Transgenic

## Abstract

Half of the global human population is dependent on rice as a staple food crop and more than 25% increase in rice productivity is required to feed the global population by 2030. With increase in irrigation, global warming and rising sea level, rising salinity has become one of the major challenges to enhance the rice productivity. Since the loss on this account is to the tune of US$12 billion per annum, it necessitates the global attention. In the era of technological advancement, substantial progress has been made on phenomics and genomics data generation but reaping benefit of this in rice salinity variety development in terms of cost, time and precision requires their harmonization. There is hardly any comprehensive holistic review for such combined approach. Present review describes classical salinity phenotyping approaches having morphological, physiological and biochemical components. It also gives a detailed account of invasive and non-invasive approaches of phenomic data generation and utilization. Classical work of rice salinity QLTs mapping in the form of chromosomal atlas has been updated. This review describes how QTLs can be further dissected into QTN by GWAS and transcriptomic approaches. Opportunities and progress made by transgenic, genome editing, metagenomics approaches in combating rice salinity problems are discussed. Major aim of this review is to provide a comprehensive over-view of hitherto progress made in rice salinity tolerance research which is required to understand bridging of phenotype based breeding with molecular breeding. This review is expected to assist rice breeders in their endeavours by fetching greater harmonization of technological advances in phenomics and genomics for better pragmatic approach having practical perspective.

## Introduction

While half of the global human population is dependent on rice (*Oryzae sativa* and *Oryza glaberrima*) as a staple food crop, there is more than 25% demand-supply gap to feed the global population by year 2030 which is a global challenge (3000 Rice Genomes Project 2014). About 20% of irrigated lands in the world are conjectured to be adversely affected by excessive salts (Pitman and Lauchli 2002). This situation presents harsh environment for crop growth and survival. The occurrence of salts in soil and water poses a serious constraint to agricultural productivity especially in those areas where irrigation is essentially required. Agricultural salinity affects about 1000 million hectares land globally which limits agricultural production and productivity of these areas. Approximately 100 million ha in South and South-east Asia which are predominantly populated are covered by problem soils where rice (*Oryza sativa*) is the staple crop. In India, about 6.73 M hectare land is afflicted with salts encompassing sodicity, inland and coastal salinity (Sharma et al. 2004). Every year, an average of 2000 million hectare irrigated land gets degraded by salt across 75 countries of the world as reported in Economics of Salt-Induced Land Degradation and Restoration (unu.edu/media- relations/releases) report (Reddy et al. 2017). There is up to 70% decrease in yield of crops like wheat, maize, rice, and barley due to salinity stress (Acquaah 2007). It is estimated that cost of such loss in crop productivity is to the tune of US$12 billion per annum globally with upward trend (Qadir et al. 2008).

Looking at the importance of rice as a food for the world population, United Nation declared the International Year of Rice way back in 2004 itself. Due to its adaptation and evolution, especially in coastal saline and sodic soils, rice has acquired many advantageous attributes (Singh 1998; Tyagi 1998). Due to increase in demand of agricultural production in last 4 decades, there is increase in irrigated land by 300%. This has increased soil salinization adversely affecting crop yield (Poustini and Siosemardeh 2004). The existing variability among salinity tolerant varieties can be utilized to further improve tolerance by their trait screening and breeding. Global warming and rising sea level have led to further increase in salinity, especially in rice grown regions by intrusion of sea salinity into inland water of coastal areas (Wassman et al. 2004; Vineis et al. 2011). Harnessing the potential of available technological leads for the management of salt affected areas can play a significant role in increasing and sustaining the food security. Such approach requires improvement of salinity tolerance in rice by best use of phenomics and genomics approaches.

Earlier reviews of salinity tolerance in rice covered Quantitative trait loci (QTL) and genes associated with tolerance and their prospects for use in marker assisted breeding (Flowers et al. 2000; Bohnert et al. 2006; Blumwald and Grover 2006; Ismail et al. 2007; Ashraf and Foolad 2013; Kumar et al. 2013; Kaur et al. 2016; Shi et al. 2017). However, there is hardly any comprehensive holistic review covering basics of phenotyping/screening, critically required for practically useful varietal improvement along with modern genomics based approach to dissect QTL into Quantitative Trait Nucleotide (QTN) by transcriptomics and GWAS approach including genome editing. If there is a critical review on these aspects of rice salinity tolerance, global community can have a more comprehensive view, which is required for rice salinity research. Looking at the cost (US $ 50–900 million) and time of at least 10 years required for development of salinity tolerant varieties by conventional breeding (Alpuerto et al. 2009), supplementation by marker assisted breeding can reduce the time from 3to 6 years. For a better return to such huge investment, it would be pragmatic to have a comprehensive review of earlier work along with available opportunities of genomic technology especially in the practical perspective.

The present review offers exhaustive and exclusive account of conventional breeding vis-à-vis modern genomics options for improved salt tolerance in rice which is genomically the most explored and economically the most important cereal crop. It endeavours to retrospect, introspect and project the strategies aimed at realistic and faster utilization of marker assisted breeding (MAB) and other genomics based technologies for improvement of salt tolerance in rice. The take away conclusions from this paper may also facilitate and direct the proper allocation of research investments for making practical impacts in this domain. In the past, the genomics and computational biology for salt tolerance have been presented and documented in isolation from real field requirements and applicability under salt affected conditions. The genomic and transgenic technologies have so far provided little success in developing successful salt tolerant rice varieties for actual field conditions. Similarly, germplasm evaluation and varietal development through field evaluation and novel phenotyping protocols for the target soil stress factors have not taken the expected advantage of the spiralling developments in molecular and computational biology. This review uniquely attempts to bridge the gaps between these two aspects for better synergy and technological benefits for practical gains.

### Benefits of Salt Tolerant Rice Varieties

Reclaiming problem soils by chemical amendments and drainage interventions is one suitable approach. However this option invariably involves higher costs which are generally beyond the economic access of poor and marginal farmers inhabiting such areas. Moreover, such pilot projects are often operative in larger community areas and individual farmers with smaller land holding size may not be able to adopt this option in a participatory manner. Identification of suitable sites for disposing off the drained-out saline water to other new areas could be another difficulty. These situations are compelling the human efforts to explore, collect, evaluate and develop salt tolerant genetic resources of the potential crops. In view of this, there remains a great possibility of selecting and breeding the salt tolerant “types” within a crop to adapt to such unfavourable conditions. This approach is well proven, simple and economical to adopt and also prevents environmental degradation. There could be a third approach i.e. synergistic approach which is based on harnessing the synergies between the environment modifying technologies and genetically enhanced plant types. This approach is considered to be more practical, economically viable, less pollution causing and efficient with tremendous potential.

### Extent of Intra- and Inter-Specific Variability for Salt Tolerance in Rice

Maas and Hoffman (1977) categorized different crops in 4 classes (tolerant, moderately tolerant, moderately sensitive and sensitive) based on the performance of one or few genotypes/varieties. For rice, soils with ECe exceeding 4 dS m^− 1^ are considered moderately saline, while more than 8 dS m^− 1^ are highly saline. Similarly, soil pH in the range of 8.8–9.2 is considered normal, 9.3–9.7 as moderate sodic, and 9.8 and above as high sodic stress. Rice is generally considered to be a sensitive crop to salinity, the threshold limit being around 3 dS m^− 1^. However, their conclusions for individual crops possibly rallied around few genotypes under their study which did not take into account the tremendous intra-specific variation and immense potential for breeding varieties with higher tolerance limits. This fact is substantiated by the study conducted by Gupta and Sharma (1990) for the response of crops to sodicity expressed in terms of exchangeable sodium percentage (ESP). Distinct genotypic differences were found in rice for threshold ESP (ESP_t_) as well as ESP at which the yield gets reduced to 50% (ESP_50_). In their study, salt tolerant rice variety CSR 1 was found to survive better with an ESP_t_ of 20.63 while MI 48, a sensitive genotype, with an ESP_t_ of 8.02 whereas corresponding values of ESP_50_ for these varieties were 81.6 and 30.6 respectively. This study clearly brought out the extent of intra-specific variability for salt tolerance in rice. Rice is found to have the maximum range of genetic variability for alkalinity tolerance while barley has the maximum for salinity tolerance. Based on affinity towards salts, plants are also categorized into two classes i.e. salt loving “halophytes” and salt sensitive “glycophytes” (Flowers et al. 1977; Flowers and Flowers 2005). Unfortunately, most of the cultivated terrestrial crop species belong to later class of glycophytes. However, no major practical breakthrough seems to be achieved so far for gene transfer from halophytes to glycophytes due to complex hybridization barriers, restricted recombination and expression of alien trait. Though most of the wild relatives are not reported to have higher salt tolerance than rice, few studies report deeper understanding of the biology of salt tolerance in a halophytic tetraploid rice wild relative *Porteresia coarctata* (Sengupta and Majumdar 2010) and successful development of inter-generic hybrids and their derivatives after its hybridization with rice (Jena 1994).

### Salinity Tolerance Parameters for Rice Phenotyping

An ideal and representative phenomics simulating natural occurrence of salts under field conditions is of crucial importance for reaping benefit of any genomics program. Phenomic data can furnish a better genotype–phenotype map, thus can decipher molecular pathways along with key candidate genes to connect genotypes to phenotypes. Phenomics must be treated as necessary and integral component of genomics in practical perspective (Houle et al. 2010). Genotype-phenotype knowledge gap can be bridged by new phenomics based on the state-of-art protocols. Metabolic pathways and intracellular regulatory networks affecting trait of interest of a given crop is a reflection of physiological and biochemical phenotype. To reap the benefit of genomics based technology, a high-dimensional physiological phenotyping is need of the hour in high-throughput mode (Großkinsky et al. 2015).

The more the intimate relationship between the morphological, physiological or biochemical trait in phenotyping with final end product yield, the more indispensable it would be for any successful mapping and QTL transfer programme. In rice salinity phenotyping related with yield, there is unfortunately, no single clear cut trait or criterion. In fact, the germplasm tolerant to salinity is also tolerant to sodicity or vice versa, yet few exceptions cannot establish this as a rule. The major visible phenotypic symptoms include leaf yellowing tip burning or panicle whitening and withering (salinity), leaf browning and necrosis (sodicity), stunted plant growth, reduced tillering, spikelet sterility, low harvest index, less florets per panicle, less 1000-grain weight, low grain yield, altered flowering duration, leaf rolling, white leaf blotches, poor root growth and patchy growth in field. Often the symptoms of salt stress also find overlap with those of other abiotic stresses, therefore their diagnosis needs careful attention. Following morphological and physiological parameters are considered for salt tolerance phenotyping in rice:

#### Agro-Morphological Parameters

##### Plant Survival (%)

Plant establishment and survival constitute a direct measure of tolerance at seedling as well as adult stages. Under moderate stress, plant survival is not a problem but under severe stress, it is a simple and better selection criterion to select a few, but robust tolerant segregates from large populations or lines from large collections.

##### Injury Score

Individual plant or group of genotypes are scored usually on 1 to 9 (1, 3, 5, 7, 9) scale where odd number lower score (1) indicates tolerant and higher score (9) denotes sensitive genotypes.

##### Phenotypic Expression

Yellowing and excessive tip burning especially in younger leaves, spikelet tip whitening and withering, stunted growth and vigour are associated traits for judging the overall phenotypic expression of the genotype.

##### Root/shoot Growth

Salt stress severely hampers the root/shoot dry and fresh weights. In rice, salt stress affects these traits but genotypic differences are also observed for the relative degree of reduction.

##### Spikelet Fertility (%)

Rice being vulnerable to salt stress at flowering stages is distinctly affected through reduced grain filling at reproductive and maturity stages. The ratio of filled grains to total number of grains is used to calculate spikelet fertility.

##### Grain Yield

It is the major selection criterion of practical importance which can be used for phenotyping the mapping populations and core collections. Generally, in the absence of any simple and reliable selection criteria, grain yield performance of the genotypes remains the ultimate and unequivocal measure of tolerance.

#### Salt Stress Indices

##### Stress Susceptibility Index

Stress susceptibility index is perceived as a better criterion of adjudging the tolerant genotypes than the per se stress performance of genotype alone because it accounts for the differences in yield potential as well. Tolerance to salt stress can be worked out by stress susceptibility index (S) on the principle of yield minimization under stress compared to non-stress environment (Fischer and Maurer 1978). The S values for individual genotype are calculated as:

$$ S=\frac{1-{\raisebox{1ex}{${Y}_s$}\!\left/ \!\raisebox{-1ex}{$Y$}\right.}_p}{D} $$

where S = Stress susceptibility index, Ys = Mean grain yield of a genotype under stress, Yp = Mean grain yield of the same genotype under non stress.

D = Stress intensity, which is calculated as under:

1-(Xms/Xmp), where Xms is the mean stress yield of all genotypes and Xmp is the mean non stress yield of all genotypes.

The higher value of ‘S’ means more stress susceptibility and lower value of ‘S’ denotes better tolerance.

##### Stress tolerance index

The stress tolerance index (STI) has been proposed by Fernandez (1992) from the yield measurements and is calculated as:

$$ STI=\left({Y}_p\times {Y}_s\right)/{\left({X}_p\right)}^2 $$

Where Y_p_ and Ys denote the grain yield of a genotype under non stress and stress conditions, respectively and X_p_ is the mean yield of all genotypes under non stress conditions. Higher STI means better tolerance and vice versa*.*

##### Mean productivity

Mean productivity (MP) for each genotype is calculated (Rosielle and Hamblin 1981) as per following formula:

$$ MP=\left({X}_s+{X}_p\right)/2 $$

Where, X_s_ = Yield of a given genotype in a stress environment, X_p_ = Yield of the same genotype in the non-stress environment.

##### Tolerance index

Tolerance index (TOL) for each genotype is calculated (Rosielle and Hamblin 1981) as given below.

$$ \mathrm{TOL}=\left({\mathrm{Y}}_{\mathrm{p}}-{\mathrm{Y}}_{\mathrm{s}}\right) $$

Where Y_p_ = Potential yield of a given genotype in a non-stress environment, Y_s_ = Yield of the same genotype in a stress environment.

##### Geometric mean productivity

Geometric mean productivity (GMP) for each genotype can be calculated (Fernandez 1992; Schneider et al. 1997) as per following method:

$$ \mathrm{GMP}=\surd \left({\mathrm{Y}}_{\mathrm{s}}\times {\mathrm{Y}}_{\mathrm{p}}\right) $$

Where Y_p_ = Potential yield of a given genotype in a non- stress environment, Y_s_ = Yield of the same genotype in the stress environment.

Higher values of MP, TOL and GMP indicate better tolerance and vice versa*.*

The above stress indices have been used by our group for phenotyping of mapping populations and breeding material for tolerance to salinity as well as sodicity (Pandit et al. 2010; Ali et al. 2013; Krishnamurthy et al. 2014, 2016, 2017; Tiwari et al. 2016).

#### Physiological and biochemical parameters

Several physiological mechanisms are perceived to contribute to the overall ability of rice plant to cope up with excess salts (Yeo et al. 1990; Flowers 2004; Blumwald and Grover 2006; Ismail et al. 2007). Physiological mechanisms that confer tolerance include Na exclusion, tissue tolerance, low Na/K ratio, efficient salt partitioning ability within plant to retain harmful salts in functionally less active organs like vacuoles and older plant parts (Singh et al. 2004). Besides Na and K, other plant nutrients like Zn, Mn, Fe, Cu and P etc. have also immense role to play in offsetting the harmful effects of sodicity in rice (Qadar 2002).Bio-chemically, esterase isozyme pattern, glyoxalase, trehalose phosphate synthase phosphatase (TPSP), increase of non-toxic organic compatible solutes like abscisic acid, proline, ethylene, glycine betaine, sugars and polyamines etc. are reported to be affected under salt stress (Singh and Mishra 2004). The mechanism of overexpression of inositol- a ubiquitous 6-carbon cyclohexane hexitol and its methylated derivative pinitol have been reported to be associated with salt tolerance in a halophyte and wild relative of rice *Porteresia coarctata* (Sengupta and Majumdar 2010). Kumar et al. (2013) have excellently reviewed the past works on rice functional genomics for the regulatory mechanisms which includes associated genes and networks for synthesis of osmoprotectants (proline, glycine betaine, TPSP, myo-inositol and fructans etc.), signalling molecules (Ca, abscisic acid, jasmonates, brassinosteroids) and ion transporters which are reported to regulate the salt stress response in rice. Since earlier results showed that no single mechanism could confer the absolute tolerance, pyramiding genes for diverse physiological mechanisms into one genetic background through marker assisted breeding holds lot of promise. A wide spectrum of germplasm in rice has been evaluated and categorized based on tissue tolerance, Na exclusion, K uptake, Na/ K ratio and reproductive stage tolerance for their use as donors for strengthening breeding program for improvement of salt tolerant varieties (Singh et al. 2004, 2010). Classifying these genotypes or donors for salinity tolerance mechanisms, inter-mating of the genotypes with high degree of expression of the contrasting salinity tolerance mechanism and identifying/screening of the recombinants for pooling of the attributes should be followed to further raise the level of salt tolerance. Crossing between the parents/donors possessing contrasting physiological traits like exclusion of Na and Cl, preferential K uptake and tissue tolerance to Na^+^, to pyramid the genes for salinity tolerance into one agronomically superior background with an objective to attain overall tolerance is the objective of such breeding programs.

Salinity causes three main effects on plants: lowering of water potential, direct toxicity of the absorbed Na and Cl and impaired uptake of essential nutrients (Flowers and Flowers 2005). Munns and Tester (2008) have opined that plant adaptations to salinity are of three distinct types: osmotic stress tolerance, Na or Cl exclusion and tolerance of tissue to accumulated Na or Cl. Depending upon the stress severity, salinity reduces growth through osmotic stress, increases cellular Na and Cl contents and exerts negative imbalance of K, Ca and NO_3_ nutrition (Flowers and Colmer 2008). The attribute of salt tolerance is governed by different physiological mechanisms and genes (Ren et al. 2005; Ismail et al. 2007; Rahman et al. 2016) and is thus amenable to genetic improvement (Gregorio et al. 2002; Flowers 2004; Singh et al. 2010). Singh et al. (2004) have extensively reviewed the progress and proposed the future thrust areas of harnessing salt tolerance in enhancing actual agricultural productivity especially in the perspective of sodic soils. Various studies have illustrated that salt tolerance is controlled by multitude of mechanisms like Na exclusion, K mining ability, lower Na/K ratio, low Cl uptake, tissue tolerance, better cellular compartmentation and higher growth vigour etc. and tolerance being believed to be growth stage-dependent (Tester and Davenport 2003; Munns 2005; Singh et al. 2008). Recently a better methodology of pruning older leaves with a view to push up Na ions into younger and flag leaves of plants subjected to salinity stress has been suggested as a better criterion for judging reproductive stage tolerance of mapping populations and germplasm lines (Ahmadizadeh et al. 2016).

### Genetic mechanisms of salinity tolerance

It has been revealed at molecular level that in plants exposed to saline conditions the key transport systems involved in ion homoeostasis are regulated by the salt overly sensitive (SOS) signal pathway (Hasegawa et al. 2000; Zhu 2000). Shi et al. (2000) and Qiu et al. (2003) found that **t**he *SOS1* (plasma membrane Na^+^/H^+^antiporter)-aided Na^+^ efflux from the cytosol is regulated by *SOS2*, a serine/threonine protein kinase which also regulates vacuolar Na^+^/H^+^ antiporter-mediated Na^+^ sequestration into the vacuole. Liu et al. *(*2012) unravelled that a salt-inducible *AP2/ERF type TF* gene, *OsERF922–ox* rice showed decreased tolerance to salt stress with an increased Na^+^/K^+^ ratio in the shoots**.** A total of 10members of *SnRK2* family were found activated by hyperosmotic stress through phosphorylation (Kobayashi et al. 2004) out of which*SAPK4* has been indicated to play an instrumental role in the salt tolerance due to decreased Na^+^ concentration in the cytosol. Similarly, Martinez-Atienza et al. (2006) also detected a plasma membrane Na/H exchanger (*OsSOS1*) gene which is functional counterpart of the Arabidopsis *SOS1* protein.

Senadheera et al. (2009) reported that as a result of salt stress, genes encoding aquaporin, a silicon transporter and N transporters were activated in the 2 contrasting genotypes i.e. FL478 (salt tolerant line carrying *SALTOL* QTL for lower Na uptake) and IR29 (sensitive). Furthermore, the transcripts for cation transport proteins like *OsCHX11, OsCNGC1, OsCAX*and *OsTPC1* showed differential regulation between these contrasting genotypes which could become future molecular targets for understanding and improving salt tolerance. The protein changes due to salinity stress were noticed in the roots of a salt-sensitive rice cultivar wherein eight proteins were induced with partia1 sequences of one with a molecular mass of 15 kilodaltons and an isoelectric point of 5.5 (Claes et al. 1990). In their study, *salT*mRNA accumulated very rapidly in sheaths and roots from mature plants and seedlings upon treatment with Murashige and Skoog salts (1%), air drying, abscisic acid (20 pM), polyethylene glycol (5%), sodium chloride (l%), and potassium chloride (1%). In general, no protein induction was observed in the leaf lamina even after the salt stress affected all parts of the plant uniformly.

Through comparative proteomics Ruan et al. (2011) identified a rice cyclophilin gene, *OsCYP2* that conferred salt tolerance in transgenic rice seedlings when overexpressed. Xu et al. (2013) also found a novel rice calmodulin-like gene *OsMSR2*, conferring enhanced salt tolerance in rice and this gene function was unravelled through the expression pattern and effects of overexpression of *OsMSR2* on salt stress. Strong and rapid induction of *OsMSR2* gene expression under salt stress was further regulated by the accumulation of proline and soluble sugar and the electrolyte leakage in rice under salt stress.

Interestingly a novel gene *OsMYB2* getting overexpressed in transgenic rice conferred triple tolerance to salinity, drought and cold stresses without hindering the growth rate compared with control (Yang et al. 2012). In rice, Hoshida et al. (2000) examined the effect of increased photorespiration on the salt tolerance by overexpressing chloroplastic glutamine synthetase (GS2) gene wherein GS2-ox rice showed increased salinity tolerance by retaining more than 90% activity of photosystem II. Jan et al. (2013) observed an increased level of expression of a number of stress-related genes, including *OsDREB2A, OsP5CS, OsProT,* and *OsLEA3* in the transgenic rice. In another case, overexpression of *OsWRKY13* gene decreased the salt tolerance through antagonistic inhibition of *SNAC1* in rice revealing that *OsWRKY13* is a negative regulator of salt stress response (Qiu et al. 2008). In rice, 10 members of *SnRK2* family were found activated by hyperosmotic stress through phosphorylationwherein*SAPK4* seemed to play a role in the salt stress tolerance via reduced Na^+^ accumulation in the cytosol (Kobayashi et al. 2004). The vacuolar Na^+^/H^+^antiporter gene, *OsNHX1* got less expressed in the transformed plants, indicating the restricted Na^+^ influx due to exclusion mechanism rather than vacuolar sequestration of the Na^+^ ions (Diedhiou et al. 2008). Through BLAST search and sequencing in Pokkali rice, Kumar et al. (2012) identified a candidate gene named as mannose-1-phosphate guanyltransferase (*OsMPPg1*) which got overexpressed in specific tissues like roots, leaves, short apical stems and panicles following the exposure to multiple abiotic stresses including salinity.

### Phenomics of rice salinity tolerance

For knowledge discovery and its applied use in rice salinity improvement breeding program, it is critical and imperative to have precise, efficient, reproducible and representative phenotyping techniques for screening of germplasm. Each of these protocols have strengths as well as limitations and therefore depending upon the specific requirements and available resources, a combination of methodologies is considered for evaluation. There are two major approaches of salinity screening, namely, invasive and non-invasive. It is also important to identify genomic regions based on phenotyping for salt stress indices like stress susceptibility index and stress tolerance index which account for minimal yield reduction under stress compared to normal conditions (Pandit et al. 2010; Ali et al. 2013; Krishnamurthy et al. 2016) rather than stress performance alone.

#### Invasive approach

Among invasive approaches, at least six different techniques are used to generate phenomics data for association studies, QTL mapping and variety improvement program. These techniques have widely been used for genes/QTLs mapping and introgression of salt tolerant genes in rice varietal improvement (Gautam et al. 2014a, 2014b).

##### Saline hydroponics

In this method, rice seedlings are planted in artificially prepared nutrient solution and pH~ 4.5 is maintained (Singh et al. 2004). Salt is added in solution to create desired level of salt stress. This method has advantages of even distribution of salts for uniform screening, screening at seedling stage at any time under controlled conditions and scope of screening large number of lines very efficiently with rapidity. However this methodology has limitations also because the salt constitution and ambient conditions of naturally salt affected soils can be exactly mimicked. Besides this, there are further limitations like feasibility of screening confined to seedling stage only and such methodology is relatively technical and costly.

##### Metal trays

For this approach of phenomics data generation, rectangular metallic trays are used which are filled with salt stressed soil for planting the seedlings. Advantages of this methodology are better control of preliminary screening at seedling stage at any time, control over desired level of stress and large number of lines can be screened rapidly. However this technique has limitation because adequate and natural soil depth is not fully ensured. Moreover, data generation is confined to seedling stage from limited space only.

##### Porcelain pots

In this, round porcelain pots of 20 or 30 cm diameter, with a capacity of 8 or 16 kg soil are used for screening. Screening can be done at seedling stage. Advantageously, here we can control the level of salt stress along with rapidity and high number of lines. Plants can be easily uprooted at desired growth stage for physiological/nutrient studies. However, micro-environment uniformity may be compromised while screening large number of genotypes in different pots.

##### Micro-plots with roof

In this method, micro-plots are constructed through reinforced concrete cement (RCC) walls and floors having dimension of about 6 m (length), 3 m (width) and 1 m (depth). These tanks are filled with normal soil which is made artificially sodic or saline. Such method has advantage of being “hybrid approach” having efficient local control along with distribution of targeted amount of salts in a plot. Besides this, it has advantage of much more closer simulation of natural conditions with desired salt concentration which is not diluted by rainfall due to translucent sheet covered roof and keeping the sides open to allow natural sunlight on the crop. However, the soil to be added in these microplots from external source should have natural nutrient composition with respect to crop requirement. Further it can be effectively used for screening large number of lines for reproductive stage tolerance due to more depth of soil. Some institutions in India like ICAR-CSSRI, Karnal, NRRI, Cuttack, CCARI, Goa and CIARI, Port Blair have established state-of- art micro-plots facility for rice salinity work. Though, establishing such facility requires more initial investments, it is worth-investing.

##### Natural fields

Here, the paddy seedlings are planted in natural sodic /saline soils. Alternatively, the normal soils are also subjected to irrigation with sodic or saline water. This method has advantage like screening of large number of lines in larger plots/longer rows under natural target conditions upto reproductive stage. This methodology has a great advantage in seed harvesting of salt tolerant lines from bigger plots for better estimation. However, this methodology has limitation of compromising spatial field variation for salts which often mars the quality and effectiveness of screening because there is no control over salt stress levels and rains etc.

##### Farmers’ fields

In this method, the paddy seedlings are planted in natural sodic /saline soils at farmers’ fields under their own management. The farmers are involved in variety selection and ranking (Gautam et al. 2014a, 2014b; Singh et al. 2014). This method has an advantage of its effectiveness for large number of lines in larger plots conditions upto reproductive stage. Besides this, it has further advantage of seed harvesting from lines along with feedback of farmers. This enables to assess impact of varietal adoption and adaptation under actual target conditions which also takes into account the farmers’ preference traits other than salinity tolerance alone. However, farmers’ fields are prone to less supervision control and there could be crop damage by stray animals, rodents and birds etc. Moreover, getting farmers’ fields of desired salt stress is sometimes a practical difficulty.

#### Non-invasive approach

In non-invasive approach, high tech image based and high throughput technologies have been recently deployed to phenotype germplasm for salt tolerance (Campbell et al. 2015; Al-Tamimi et al. 2016). In conventional methods of salt stress induced phenotyping, physiological parameters such as stomatal conductance, osmotic potential, dark-adapted quantum yield and biomass allocation are recorded. This manual method is time consuming, labour intensive and destructive in nature. Modern phenotyping based on high tech imaging system has advantage of high throughput, less time consuming, higher automation with higher accuracy (Awada et al. 2018). Such machine based high-end phenomics accelerates selection of plant varieties which can perform relatively better in the field with more reproducibility when affected by salt stress (Siddiqui et al. 2014). Since root is the first tissue to sense salinity thus phenome data of root system architecture generated by non-invasive X-ray tomography can be used to study genotype and environment interaction. (Rogers et al. 2016).

Besides this, such new Phenomics methods are also required as salinity tolerance mechanism and indicators vary across between tissues as well as varieties. Thus it is imperative to have non-invasive stage- and tissue- specific accurate data for advance genomics based association studies. Salinity response in rice has two phases. In shoot ion-independent ‘osmotic stress’ phase, there is stomatal closure with increase in leaf temperature as well as inhibition of shoot elongation. In shoot ion dependent ‘ionic phase’, there is growth inhibition and senescence of older leaves (Reddy et al. 2017). This has been clearly demonstrated by specific experimental studies having non-destructive image-based phenotyping of salt tolerance traits of two rice varieties (IR64 and Fatmawati) and phenomic data of shoot area (Reddy et al. 2017). High precision non-destructive approach offers accurate “new phenomics data” with better repeatability in real time mode which can be used to decipher genetic basis of salinity tolerance mechanisms required in pyramiding of different tolerance mechanism in the targeted genetic backgrounds.. Different levels of salt stress can be quantified by spectral imaging over time by total area of shoot and senescent part, obtained by images in RGB (red-green-blue) and fluorescence mode (Hairmansis et al. 2014).

Different spectra of imaging can be used for generation of tissue specific phenomic data, for example, root and shoot imaging can be done by near infra-red and visible colour range at desired angle in timeline. A phenotypic data can be generated by image processing to correlate with various traits/ parameters for growth rate, biomass, photosynthetic and transpiration use efficiency (TUE), concentration of metabolites in response to differential salinity stress in timeline. Phenotype genotype interaction model can be generated using different software to measure the productivity parameters of rice in response to salinity (http://www.iari.res.in/files/Latest-News/PlantPhenomicsCentre_inauguration_News_13102017.pdf). Such high throughput phenotyping (HTP) has been found successful in rice phenomics data generation for GWAS studies. In such work, phenotypic data are generated by canopy level sensors having high precision real time kinematic GPS controlled movement. Here, 3-band reflectance sensor, namely, infrared canopy temperature sensor, ultrasonic canopy height sensor and spectral reflectance bands of near infrared (760–800 nm), red edge (730 nm) and red (670 nm) are used. Such system of phenomics also records air temperature, humidity and photosynthetically active radiation measurement (Tanger et al. 2017). Artificial intelligence like deep learning techniques have been used for plant stress (biotic and abiotic) phenotyping for identification, classification, quantification, and prediction (ICQP) to make best use of digital image–based phenomics data (Singh et al. 2018). Such technique based panicle segmentation algorithm has been found promising in rice to study different reproductive stage in different field environment for non-destructive yield estimation (Xiong et al. 2017).

Rice plant copes up with salt stress by 3 distinct mechanisms, (i) osmotic tolerance, (ii) exclusion of sodium ion from shoot and (iii) tissue tolerance. Osmotic and tissue tolerance being dynamic processes require real time, uninterrupted and non-destructive measurements. Because of this, automated imaging systems are imperative and efficient for the measurement of salinity effects (Berger et al. 2012). Using contrasting genotypes, non-destructive image-based phenotyping protocols have been very promising for screening rice for salinity tolerance with advantage of specificity, accuracy and time. Such advanced phenotyping protocols are now available in many part of the globe, for example Australian Plant Phenomics Facility (http://www.plantphenomics.org.au), European Plant Phenomics Network (http://www.plant-phenotyping-network.eu) (Hairmansis et al. 2014), UK (https://www.plant-phenomics.ac.uk/), India (at ICAR-IARI: http://www.iari.res.in/files/Latest-News/PlantPhenomicsCentre_inauguration_News_13102017.pdf; ICAR-NIASM: http://www.niam.res.in/Phenomics-facility; ICAR-IIWBR: http://www.iiwbr.org/phenotyping-facility/). Drone based field data observation can further enhance both precision and speed of phenotyping with lot of cost reduction.

Since genetic basis of salinity tolerance is polygenic and is mediated by myriad of physiological responses thus non-invasive high throughput real time spectral imaging can be used for association studies of physiological and biochemical traits/events. Such approach in rice for salinity response study has revealed genomic regions on different chromosomes and their association with different time and dose lines of salinity. For example, chromosome 3 and chromosome 1 are strongly associated with early growth response and controlling ionic stress at early growth stage by change in fluorescence shift, respectively (Malachy et al. 2015). Thus it can be concluded that both invasive and non-invasive approaches have their specific advantages and disadvantages with respect to spatial variability of salt distribution, precision control and monitoring in hydroponics and natural soil system, weather interference, rainfall control, soil and water medium, appropriateness for seedling, reproductive and seed harvesting stage etc. Therefore, a particular phenotyping methodology can be opted as per the requirement, genetic nature and quantum of the breeding lines, technical and financial feasibility besides the scale and rapidity of screening.

### QTLs/ genes for salt tolerance and their database

Through an F_2_ population derived from salt tolerant mutant and sensitive genotype, Zhang et al. (1995) found that enhanced salt tolerance was governed by a major tolerant gene which showed incomplete dominance. By using a doubled haploid population Prasad et al. (2000) mapped 7 QTLs for tolerance to salinity stress at seed germination and seedling stages. Koyama et al. (2001) revealed that the QTLs for Na and K uptake were found on different rice chromosomes. Lin et al. (2004) through a cross Nona Bokra (salt tolerant) x Koshihikari (sensitive) varieties detected 3 QTLs on chromosomes 1, 6 and 7 accounting for the number of survival days of seedlings under salt stress. Later in the same mapping population, Ren et al. (2005) discovered a QTL *SKC1* accounting for about 40% phenotypic variation in shoot for the K mining ability under salt stress. Takehisa et al. (2004) also reported QTLs on chromosomes 2, 3 and 7 for stable tolerance to saline flooded conditions through backcross-inbred lines derived from Nipponbare (moderately salt-tolerant variety, as recurrent parent) and Kasalath (salt sensitive). From the mapping population derived from salt-tolerant japonica rice (Jiucaiqing) and sensitive *indica* variety (IR26), Wang et al. *(*2012) mapped 6 large effect QTLs and concluded that one QTL caused decreased Na^+^ concentration in shoots which could be a strong candidate gene for marker assisted selection. Lee et al. (2006) located two QTLs viz. *qST1* and *qST3* respectively on chromosomes 1 and 3 for seedling stage tolerance through RILs developed from Milyang 23 x Gihobyeo cross.

Ammar et al. (2007, 2009) mapped a major QTL for multiple salt tolerance parameters on chromosome 8 and three other major QTLs for Cl ion concentration through F_2–3_ mapping population derived from CSR27 (tolerant) X MI48 (sensitive) cross. Subsequently through RILs derived from the same population, 8 significant QTLs were mapped on chromosomes 1, 8 and 12 including an important QTL for higher spikelet fertility at reproductive stage salt tolerance on chromosome 8 (Pandit et al. 2010). In another study, five major QTLs with considerable effects for root and shoot traits under salt stress were reported (Sabouri and Sabouri 2008). Ahmadi and Fotokian (2011) identified a major QTL on chromosome 1 conferring higher K^+^ mining ability under salt stress. Ghomi et al. (2013) conducted the QTL analysis of physiological traits related to salt tolerance using F_2:4_ population developed from a cross between a tolerant variety (Gharib) and a sensitive variety (Sepidroud) and reported 41 QTLs for 12 physiological traits under salinity stress. In other studies, many new QTLs for seedling stage tolerance have been mapped in rice (Alam et al. 2011; Lee et al. 2007; Pushparajan et al. 2011). Through an association mapping involving 347 global rice germplasm lines, Cui et al. (2015) discovered a total of 40 markers of which 25 and 15 were associated with tolerance to salinity and alkalinity, respectively wherein 3 markers were common for both salinity and alkalinity stress tolerance. Molla et al. (2015) studied a total of 220 salt responsive genes and employed 19 primer sets to detect polymorphism across tolerant and sensitive groups and revealed the utility of salt responsive candidate gene based SSR (cgSSR) markers for distinguishing tolerant and sensitive genotypes. Recently, Bizimana et al. (2017) mapped 20 new QTLs located on chromosomes 1,2,4,6,8, 9 and 12 in a novel source Hasawi, a Saudi landrace which could diversify the nature of salt tolerance. Based upon stress susceptibility indices, Shi et al. (2017) identified 11 loci on chromosomes 1,5,6,11 and 12 containing 22 important SNPs conferring tolerance at seed germination stages and concluded that japonica types have better salt tolerance than *indica* types. Regarding wild relatives of rice, a study conducted in *Oryza rufipogon* identified four QTL clusters located on chromosomes 6,7,9 and 10 explaining 19 to 26% phenotypic variation for root and shoot traits under salt stress (Tian et al. 2011). Kaur et al. (2016) have performed a meta-analysis of many known genes for controlling salt tolerance in rice to prioritize candidate genes. In our overall compilation, maximum number of salt tolerance associated QTLs are reported on chromosome 1, followed by 3, 4, 6, 7, 2 and 9 (Additional file 1 Table S1).

There are several available QTL databases of rice and rice informatics. One of the major plant QTL databases, Gramene-QTL (Ni et al. 2009) records more than 8000 QTLs of rice along with their chromosomal distribution and various trait association but there is no comprehensive database specific to the rice salinity associated QTLs. Even the rice species specific database like, Q-TARO: QTL Annotation Rice Online Database contains more than 1000 QTLs for 21 different traits (Yonemaru et al. 2010) but again salinity trait is limited. Even in OGRO database (Overview of Functionally Characterized Genes in Rice Online database) module of Q-TARO (http://qtaro.rd.naro.go.jp/ogro) contains 1949 entries of genes over QTL but hardly 100 entries of genes related to salinity having chromosomal positions and coordinates over genome are reported. Nagamura and Antonio (2010) reviewed the rice informatics resources having 37 web genomic resources but none of these resources are specific to rice salinity tolerance. Even in International Rice Informatics Consortium, there is no publicly accessible salinity trait specific QTL information. Another informatics resource for rice, RiceXPro Version 3.0 also does not contain rice salinity specific resource (Sato et al. 2012).

### The discovery of *SALTOL* QTL-a success story

At International Rice Research Institute (IRRI), Philippines, a major QTL called as *SALTOL* associated with seedling stage tolerance and explaining about 40% variation for salt uptake was detected on chromosome 1 through a RIL population derived from tolerant (Pokkali) x sensitive (IR29) cross (Bonilla et al. 2002). Subsequently, *SALTOL* was deeply characterized through linked SSR markers flanking the QTL region (Thomson et al. 2010). Another study revealed that within *SALTOL* QTL region in the Pokkali variety, precise salinity induced factors (SIFs) were found expressed which could be putatively associated with vegetative growth, fertility, viability and early flowering under salinity stress (Soda et al. 2013).

For *SALTOL* QTL, FL478 (IR66946-3R-178-1-1) a line developed from IR29/Pokkali population has been promoted as an improved donor for introducing salinity tolerance in rice breeding programs through marker assisted back-crossing (MABC). Using MABC, the *SALTOL* was transferred into BR11 and BRRI dhan28 in Bangladesh (Rahman et al. 2008), AS996 and BT7 in Vietnam (Huyen et al. 2012; Hien et al. 2012), Rassi in West Africa (Bimpong et al. 2016) and Pusa Basmati 1121 and PB6 in India (Babu et al. 2017; Singh et al. 2011). In India, the *SALTOL* introgression in mega rice varieties is in progress through multi-institutional programs (Singh et al. 2016; Geetha et al. 2017).

### Limitations of salinity tolerance QTLs in marker assisted breeding

Unfortunately, not a single significant QTL with large effect for rice salinity conditions with practical impact is available. Moreover, marker assisted selection and backcross breeding are considered to have limitation of higher cost, linkage drag of undesirable traits because of poor resolution of chromosome beyond few centiMorgan having QTL (Martinez et al. 2002) and statistical inaccuracy in estimating environmental and genetic background effect on the trait (Flowers 2004). Though *SALTOL* and most of other reported QTLs are effective at seedling stage, our study aimed at reproductive stage tolerance and grain yield performance under both saline and sodic stress conditions could not establish the superiority of the *SALTOL* QTL derived material compared to the conventionally developed check varieties (Ali et al. 2013). These indications call for a need to explore and utilize other QTLs with better potential especially for reproductive stage traits.

### QTLs for reproductive stage salinity tolerance

QTLs for reproductive stage tolerance to salinity have been reported by many workers (Lang et al. 2001, Pandit et al. 2010, Islam et al. 2011, Mohammadi et al. 2013, Chai et al. 2014, Hossain et al. 2015), but limited studies have been reported for sodicity tolerance (Tiwari et al. 2016). However, successful validation and transfer of these QTLs into mega varieties for practical gains are still awaited.

### QTL refinement by NGS data for fine mapping and molecular breeding

The marker assisted selection (MAS) can accelerate the speed and precision of conventional plant breeding because it is growth stage independent, unaltered by environment, free from dominance and epistatic effects and is quite effective in early segregating generations. Nevertheless, a practically useful QTL for any trait should ideally (i) have been identified through phenotyping under representative and reproducible stress conditions (ii) have large contributory effect on total trait expression, (ii) have desirable expression across different environments and genetic backgrounds (iii) be < 1 cM away from linked markers to reduce linkage drag and (iv) have additional markers identified within the QTL region for fine mapping and use in marker assisted selection.

In the process of QTL mapping, sharper the contrast between two parents for salinity/sodicity tolerance, higher will be the chances of unravelling the strong and useful QTLs. Secondly, the tolerant and sensitive parents selected for development of mapping population like recombinant inbred lines (RILs) should have preferably similar flowering/maturity times to practically facilitate advancement of all lines with equal and unbiased probability for stabilization. The precise understanding of molecular basis of salt tolerance and genomic information can lead us towards realizing the concept of “breeding by design” as advocated by Peleman and Vander Voort (2003). Here it is also important to mention that some mapping populations like RILs may not allow full detection of the useful QTLs due to small population size and presence of lesser functionally polymorphic alleles (Tuberosa and Salvi 2007). Therefore, working on multi-parental crosses of MAGIC (Multi-parent advanced generation inter-cross) population and mini core collections can also enlarge the spectrum of genetic and molecular variability to enhance the chances of mapping and utilizing functionally useful alleles. Development of NILs (near isogenic lines) each having QTL for specific physiological trait for salt tolerance in a common genetic background can be useful to quantify and harness the advantage of each component of tolerance. Since different growth stages in rice are differentially vulnerable to salinity effects (Singh et al. 2008, 2010), studies on identification of QTLs for tolerance at vulnerable stages can be given more priority. Different QTLs thus identified can be pyramided in a single recipient variety through marker assisted breeding for genetic tailoring of “super tolerance”.

### Genome sequencing and GWAS revealing rice salinity QTL into QTN

Rice is a model crop species due to its relatively smaller genome size (430 MB) but with wide genetic variability having adaptive attributes for tolerance to acidity, sodicity, salinity, nutrients toxicities and deficiencies etc. Moreover, smaller genome size facilitates genome sequencing, gene detection and transfer through molecular approaches. There is unlimited and unthinkable opportunities for genetic improvement by genomics approach (IRGSP 2005; Edwards et al. 2016). Such approach is highly promising in improvement of salt tolerance with the help of molecular markers to enhance the precision and efficiency of crop for better productivity (Jena and Mackill 2008). Genomic data of rice has been very successfully used for marker discovery like SSRs, SNPs and InDels which has been used for knowledge discovery like QTL mapping and germplasm management. For example**,** Singh et al. (2009) have validated and described a genome wide set of 436 highly variable SSR (HvSSR) markers with repeat lengths of 51–70 bp for their consistent amplification and higher degree of polymorphism. These HvSSR loci showed more than twice the level of polymorphism than random SSR markers with average repeat length of 34 bp which enable them a better choice for QTL mapping and fingerprinting studies in rice for varietal signature.

Recently, the development of high quality reference genome of rice has facilitated the high density genotyping (McCouch et al. 2016) and re-sequencing of more than 3000 rice varieties (Alexandrov et al. 2015; Duitama et al. 2015). Therefore, genetic and molecular dissection of salt tolerance and its components can pave the way for systematic and precise gene transfer in salt sensitive but otherwise superior cultivars through marker assisted breeding for large and long term impacts. Though there have been numerous attempts to review work on genes/ QTLs for salt tolerance (Ashraf and Foolad 2013; Blumwald and Grover 2006) the present review offers exhaustive and exclusive account of QTL mapping and utilization for salt tolerance, specifically in rice which is genomically the most explored and economically important cereal crop.

Using computational genomics, now it is possible to dissect QTL into genic regions along with nucleotide variants called quantitative trait nucleotide (QTN) which is responsible for variation in quantitative traits. Now it is possible to understand adaptive phenotypes along with population structure having attributes of SNPs with inheritance, allele frequencies and evolutionary dynamics (Lee et al. 2014). GWAS has advantage of computing statistical association of all nucleotide variants which are accumulated in a particular genotype due to ecological and agricultural selection to decipher phenotypically important QTNs. It is also correlated with population structure which may differ between two populations. These refined QTNs of specific traits can be used for faster and accurate selection in varietal improvement program by introgression of desirable alleles (Mitchell-Olds 2010).

GWAS based QTN mapping is more efficient than linkage mapping of QTL (Naveed et al. 2018). In a GWAS study based on mini-core (25 countries, > 200 varieties) using 700 K SNP chip of rice, 22 candidate genes and 20 QTNs have been identified which are associated with 11 different salt tolerant traits at germination and seedling stages (Naveed et al. 2018). In another study using SNP chip 50 K, a total of 6068 polymorphic SNPs were obtained in biparental population studies wherein a total of 11 desirable and 23 undesirable QTNs were reported associated with salt tolerant traits (Tiwari et al. 2016).

In GBS (Genotyping By Sequencing) approach of GWAS using 235 temperate *japonica* rice accessions, association study has been conducted with 30,000 SNP markers wherein 27 QTLs were validated along with the discovery of candidate genes related to salinity (Frouin et al. 2018).

Wild rice has been used as a source of salinity tolerant genes. For example, the salt tolerant Chinese Dongxiang wild rice has been successfully used to introgress genes conferring salinity tolerance into recipient rice variety NJ16through repeated back-crossing (Quan et al. 2018). Such resources are used for genome resequencing to get SNP and indel markers and can also be used for QTL discovery. Both approaches supplement each other by discovery of candidate genes and harbouring QTNs.

SNP and Indel discovery has been done through whole genome sequence analysis of representative salinity tolerant variety Godawee. Such variety specific whole genome data can be used to mine putative SNP and Indel markers from Salt Tolerance Related Genes (STRGs) which could be used in future association studies (Singhabahu et al. 2017). The 3 K RG 6.5 m SNP dataset (478 accessions from 46 countries) available at Rice SNP-Seek Database (http://www.oryzasnp.org/) (Alexandrov et al. 2014) has been used successfully in GWAS for revealing 22 SNP from 11 loci associated with stress-susceptibility indices (SSIs) of vigor index (VI) and mean germination time index of salinity tolerance (Shi et al. 2017). In another study of very same 3 K RGP SNP set using 203 temperate japonica rice accessions with 9 salinity traits, 26 QTLs were analysed which revealed 6 candidate genes in 11 known QTLs having SNPs. This study also reconfirmed the well-known major Saltol QTL having OS HKT1; 5 (SKC1) gene controlling Na+/K+ ratio (Batayeva et al. 2018).

It is interesting to note that earlier findings of the QTL discovery based on the statistical genetics are supplementing the findings based on GWAS. In a study based on the diversity panel of 306 rice accessions involving HD 700 K has revealed 1900 significant SNPs and ~ 2300 candidate genes involved in rice salinity tolerance. This study clearly dissected a well-known major QTL, *Saltol-1 *associated with shoot Na/K ratio located on chromosome 1 (~ 9.3 Mb – 16.4 Mb) (Bonilla et al. 2002; Soda et al. 2013) having its key candidate gene, OsHKT1; 5 (positioned at 11.45 Mb) which controls sodium ion uptake in xylem (Ren et al. 2005).

Through rice GWAS using 700 K SNP chip and HTP phenotyping, new QTLs have been discovered which are associated with flowering time, height, biomass, grain yield, and harvest index. This study demonstrated that such approach can be used to discover new QTLs having influence on yield and yield components that can be mapped non-destructively in a fraction of time. Thus HTP and GWAS can be used for efficient screening of large populations along with accuracy in breeding value prediction. Rapid and early prediction of phenotypes can enable the breeder to enter into the next breeding cycle thus accelerating rate of genetic gain per unit time which is the main endeavour of molecular breeding program (Tanger et al. 2017).

### Transgenic approach

Transgenic approach has been a powerful tool in plant breeding where genes are taken from other species in order to get insertion of gene (s) controlling traits without dilution of any desirable trait of a recipient elite genotype (Bhatnagar-Mathur et al. 2008). Transgenic approach has been used to improve salinity tolerance in rice by various approaches like control of organic solutes, antioxidants detoxifying ROS, transport of ion, late embryogenesis proteins (LEP) and heat-shock proteins, programmed cell death (PCD), signal transduction and transcription factor (TF) which are involved in coping mechanism against higher salinity.

Various novel genes have been used for development of salinity tolerant transgenic rice. These include genes which are involved in antioxidants and ROS detoxification activity such as CAT1 and GST from *Suaeda salsa* (Zhao and Zhang 2006), GlyII from *Oryza sativa* (Singla-Pareek et al. 2008), GS2 from *Oryza sativa* (Hoshida et al. 2000), katE from *Escherichia coli* (Nagamiya et al. 2007; Moriwaki et al. 2008), Mn-SOD from *Saccharomyces cerevisiae* (Tanaka et al. 1999), Sod1 dismutase from *Avicennia marina* (Prashanth et al. 2008). Similarly, genes involved in ion homeostasis and compartmentation have been used such as nhaA from *Escherichia coli* (Wu et al. 2005), AgNHX1 from *Atriplex gmelini* (Ohta et al. 2002), OsNHX1 Vacuolar from *Oryza sativa* (Fukuda et al. 2004; Biswas et al. 2015; Chen et al. 2007), SOS2 from *Schizosaccharomyces pombe* (Zhao et al. 2006), PgNHX1 from *Pennisetum glaucum* (Verma et al. 2007) and OsKAT1 from *Oryza sativa* (Obata et al. 2007). Genes involved in osmotic adjustment, such as ADC from *Avena sativa* (Roy and Wu 2001), codA from *Arthrobacter globiformis* (Sakamoto and Murata 1998; Mohanty et al. 2002), COX from *Arthrobacter pascens* (Su et al. 2006), P5SC from *Vigna aconitifolia* (Karthikeyan et al. 2011), P5CSF129A from *Vigna aconitifolia* (Kumar et al. 2010), SAMDC from *Tritordeum* (Roy and Wu 2002), TPS, TPP and TPSP from *Escherichia coli* (Garg et al. 2002; Jang et al. 2003; Joshi et al. 2019) have shown considerable success. Genes involved in programmed cell death (PCD) are also successfully reported for development of salinity tolerant transgenic rice using genes like AtBAG4 from *Arabidopsis*, p35 from *Baculovirus*, Hsp70 from *Citrus tristeza* virus and SfIAP from *Spodoptera frugiperda* (Hoang et al. 2014; Hoang et al. 2015). Even genes like CNAtrCalcineurin gene from animal (mouse) were used to get transgenic rice having enhanced salt tolerance due to less sodium accumulation in roots with growth under high sodium chloride stress.

Though lot of efforts with commercial success have been made in transgenic rice for various traits, such success in salinity tolerant transgenic rice is yet to be achieved. Most of the transgenic rice are developed in USA (11.5%), Europe (8.9%), Oceania (1.1%) and Africa (1%) but majority of the field trials are in Asian countries (77.5%), predominantly in China (47.8%) and Japan (20.2%). Among the commercialised transgenic rice varieties, herbicide tolerance and insect resistance were first. Salinity tolerant transgenic rice varieties have been developed using various genes of both ABA dependent and independent pathway genes which has been reviewed by Reddy et al. 2017. Recently, a salt-tolerant transgenic rice has been developed using over-expressing a gene, OsIF (*Oryza sativa* intermediate filament) from wild rice (*Porteresia coarctata*) which is native to India, Bangladesh, Sri Lanka and Myanmar (Soda et al. 2018). Salinity tolerant transgenic rice development is complex due to critical events like ion homeostasis and compartmentation, programmed cell death, signal transduction and transcription factors based regulations several set of genes are involved. In fact, there is no single transgene which can increase salinity tolerance in rice for the entire life cycle (Hoang et al. 2016). This issue is further compounded by specificity of the salinity tolerance genes for seedling and reproductive stages and their expression in all genetic backgrounds and above all the social, ethical and environmental concerns about transgenic technology. However, efforts have been made to dispel fears about genetically engineered crops (Pental 2019). Though transgenic rice varieties have been developed successfully, their expansion is not uniform across globe due to complex process of statutory guidelines. Being GMO, each transgenic rice variety must comply international guidelines of Cartagena protocol which is implemented by each country by its own biosafety guidelines in its territory (Pythoud 2004). Major impediments in expansion of transgenic rice are asynchronous approval and release, illegal propagation of seed, contaminated/ accidental detection in non-labelled transgenic rice produce in countries having zero tolerance (Fraiture et al. 2016).

### Genome editing approach

Genome manipulation by targeted editing of the genomes has brought revolution in plant genomics approach for improvement of traits against biotic and abiotic stresses **(**Kamburova et al. 2017; Yin et al. 2017). Genome editing can be accomplished through approaches like meganucleases, TAL effector nucleases (TALENs) and zinc finger nucleases (ZFNs) and clustered regularly interspersed short palindromic repeats (CRISPR)/Cas system (Hoang et al. 2016;Kamburova et al. 2017). Among these, CRISPR–Cas9, has widely been used to develop transformable plants by inducing mutations through non-homologous end joining of double-stranded breaks (Yin et al. 2017). This approach is effective, desirable and achievable through precise gene editing to develop salinity tolerant transformation-recalcitrant rice varieties. Unlike transgenic approach it does not involve alien gene integration and precisely incorporates DNA modification (editing) which is directed by homology-dependent repair. Being non-genetically modified approach having high precision for editing of specific genomic region for achieving the desired phenotype it is socially accepted and much more preferred over transgenics development (Kamburova et al. 2017).

Genome editing can be used with novelty in rice to make new varieties having attributes of higher yield and quality along with robust tolerance to abiotic and biotic stresses. CRISPR/Cas has been used widely in many crops and in rice also it has shown huge success in making desirable changes against various biotic and abiotic stresses (Khang 2018). There are many successful examples of gene editing in rice like genes controlling albino (OsPDS), photo-period sensitive male sterility (OsPMS3), transcription factor MYB family (OsMYB1 and 5), lethal (OsEPSPS), albino young seedling (OsYSA), pleotropic phenotype (OsMSH1), abaxial leaf rolling (OsROC5), drought tolerance (OsDERF1) and early seedling leaf chlorosis (OsSPP) etc. (Zhang et al. 2014). Recently there are successful examples of abiotic trait improvement in rice using genome editing which include enhanced cold tolerance by using TIFY1b transcription factor gene editing using Nipponbare rice variety (Khang 2018), improved blast disease resistance in rice variety Kuiku131 by targeted editing of gene OsERF922 and improvement of herbicide resistance trait by editing Acetolactate Synthase 1 (ALS1) gene (Sun et al. 2016**).** These examples clearly indicate that there is a huge potential of genome editing for trait improvement. However, more gene editing research is required specifically for enhancing salinity tolerance in rice.

Functionally relevant SNP obtained from GWAS studies can be pragmatically used for genome editing (Tak and Farnham 2015). If GWAS is applied in rice to identify so called non-phenotypic variations like eQTL (expression QTL), methylation QTL (meQTL), metabolite QTL along with novel and valuable alleles, then genome editing can be used to facilitate combination of new set of alleles (Druka et al. 2010; Vidalis et al. 2016). Such approach has potential to make paradigm shift in rice breeding especially through genomics-driven crop design.

### Soil metagenomics in salinity management

Soil metagenome can be an alternative strategy to identify and characterize salt-tolerant microbes which can promote crop growth (Dodd and Pérez-Alfocea 2012). Soil salinity tolerant microbes have been successfully demonstrated in promoting growth of many crops which can be potentially harnessed to further supplement the advantages of crop tolerance and under those conditions where salt tolerant germplasm development is a great challenge (Dodd and Pérez-Alfocea 2012). Besides conferring stress tolerance to plants, such microbes have also been found advantageous as plant growth-promoting rhizobacteria (Etesami and Beattie 2017; Etesami 2018). The rhizosphere microbiome has a key role to play during plant survival under adverse conditions. Therefore, if inventory of such soil bacteria from different saline environments is studied then it might be a source of discovery of potential inoculants and gene prospecting which can be applied in saline lands to manage or improve crop productivity (Mukhtar et al. 2018). Such culture-independent method can be used for knowledge discovery of halophiles for unique osmotolerant mechanism under salt stress. There are three classes of halophilic bacteria with range of slight (0.2–0.85 M NaCl), moderate (0.85–3.4 M NaCl), and extreme (3.4–5.1 M NaCl) saline adaptation (Ventosa et al. 2008). Such microbes constitute a potential repository of novel enzymes like proteases, xylanases, cellulases, and amylases having properties of extremophiles which can be relevant not only for soil remediation but can also be used in the industrial application (Delgado-García et al. 2014; Dastgheib et al. 2011).

Scientists have developed a wide range of methods to study microbial diversity, community structure and functions to understand plant-microbe interactions and soil biology (Rincon-Florez et al. 2013; Mukhtar et al. 2017). High throughput sequencing approaches have been developed to understand the complexity of microbial communities in a wide range of environments. Continuing advances in sequencing technology enable us to study the dynamics of microbial population by using metagenomics and meta-transcriptomics. Such approaches may uncover not only the composition of the microbiome community, but also the altered functional mechanisms of the microbes enabling them to survive under harsh conditions. NGS based approach can be used for metagenomics as well as meta-transcriptomics to have diversity profile of microbiome community along with functional traits deciphering the mechanism of survival under harsh condition ((Mukhtar et al. 2018). Such studies are not only useful for knowledge discovery of osmo-tolerance genes but they can also be used to get potential gene constructs for enhanced salinity tolerance for crop improvement (Ahmed et al. 2018).

## Conclusion and the way forward

There is an urgent need to re-orient our strategy towards practical delivery in terms of development of robust and widely adaptable salt tolerant rice varieties in light of available databases, accumulated knowledge resources and advances in both phenomics and genomics. However, harmonizing the high throughput techniques for phenomics and genomics is both a challenge and opportunity. The aim of this first ever comprehensive review is to systematically align and harmonize the past progress with current developments, available opportunities, and present a futuristic orientation for harnessing the genetic worth of salt tolerance in rice. System biology approach having convergence of phenomics, transcriptomics, proteomics and metabolomics data can be used to decipher the molecular mechanism of salt tolerance in rice varieties by discovering the key candidate genes. Phenomic analysis is the key to functional genomics. Image analysis has been used to compare shoot ion independent and dependent stress for elucidating genetic basis of physiological events. It concludes that plant physiology must be used to evaluate the function of target genes controlling salinity tolerance and such approach can bridge the gap between genomics and phenomics data. Evaluation of key candidate genes controlling salinity will be more pivotal and pragmatic in development of salinity tolerant varieties. Physiological data must be generated by non-invasive phenotyping. If a parallel set is used for invasive phenotyping to generate transcriptome and proteomics data, then function of key candidate genes can be associated to visualize “live gene function” at physiological level. Various mechanisms are involved in morpho-physiological traits associated with salinity tolerance like high shoot/root biomass, shoot Na + accumulation, shoot Na+/K+ ratio, water uptake and transpiration, stomatal transpiration. Such approach can identify the genetic basis of different salt tolerance mechanisms. Key candidate genes associated with different physiological mechanism can be used in pyramiding of different tolerance mechanisms in salinity tolerant varietal improvement. Though spectacular and phenomenal success has been obtained in development of salinity tolerant varieties by conventional breeding which has several limitations, yet there is no replacement of it. All molecular breeding programmes must supplement or complement it.

Conclusion can be drawn from this review that though spectacular knowledge and phenomenal success have been obtained in various domains related with the development of salinity tolerant varieties, there is need to fill the potential gaps. There is no replacement of the conventional breeding, but its limitations in terms of speed, accuracy and drudgery can be overcome by molecular breeding programmes. In molecular breeding for salinity tolerance, initial success has been made by the discovery of many QTLs and several rice salinity GWAS reports, but still there is a considerable gap between knowledge discovery and actual use of molecular breeding in realization of its full potential to cater to the needs for development of field oriented salt tolerant rice varieties to befittingly address the global challenge of soil and water salinity. While the conventional phenotyping and breeding approaches are sound, the advantages and opportunities thrown open by automated phenotyping should be availed for faster gains.

Since modern genotyping protocols are well developed and high throughput in rice, phenotyping models need more consideration because capturing “right QTL” largely depends upon right phenotyping. It is suggested to precisely phenotype the mapping population across diverse salinity locations and crop seasons to come out with practically useful and adaptable QTLs. Stage-specific and stress-specific QTLs may be identified for need based deployment. In this regard, the screening methodology should be simple and high throughput, reproducible and representative of near-field conditions. In summary, QTLs with maximum contribution to the overall variation for reproductive stage yield traits should be identified besides their performance validation across locations, salt stress levels and genetic backgrounds.

Marker assisted pyramiding of QTLs for different tolerance mechanisms in a popular variety can pay rich dividends. Genome editing has great commercial potential as an adjunct for rice molecular breeding not only for salinity trait improvement also for the multi-trait improvement of economically and agronomically relevant market based desirable traits like biofortification, nitrogen use efficiency, diabetic friendly rice, grain length/ texture and aroma coupled with tolerance/resistance to other abiotic and biotic stresses. In other words, marker assisted breeding should go hand in hand with market assisted breeding.

Therefore salinity improvement research must be integrated with other plant traits and market oriented factors. More global efforts are required to develop statistical models from different geo-climatic conditions to capture genotype-phenotype relationship along with development of genomic resources. Such efforts are also required for high precision and reproducible phenotyping of rice salinity tolerance which would strengthen the very age-old backbone of conventional breeding but with much accelerated pace and manoeuvrability in the desired direction than ever before by convergence of QTL into QTN and MTA of GWAS into genome edited salt tolerant rice varieties. As such information explosion requires its conversion into true knowledge having practical perspective of rice breeding in the field rather than academic and technological advancements alone.

It is opined that sustainable country-wide networks for periodic convergence are required among plant breeders, agronomists, physiologists, microbiologists and molecular biologists in a symphony, all aiming at the same goal with practical perspective. While doing so, discovery and utilization of practically relevant genes/QTLs should be accelerated to justify the research investments in the era of climate change and rising salinity. Since development of market and field problem oriented salt tolerant rice varieties through required QTLs identification and introduction are important in increasing productivity of disadvantageous degraded lands in an eco-friendly manner without additional costs on chemical amendments and drainage interventions, more societal gains are expected through research investments in this domain of crop improvement.

However, while using genome editing the potential threat to diversity sustainability by sweep-off due to “gene drive” (selective dominance of limited varieties) should not be overlooked. Global consortium for economic and efficient use of knowledge along with germplasm development is required in altruistic spirit for crop like rice which is grown under unfavourable conditions and is a grain in the bowl of poor to rich. Neither classical breeding nor the molecular breeding approach alone can be successful in development of salt tolerant rice varieties. Convergence of phenomics, GWAS, transcriptomics, proteomics and metabolomics data is warranted to fill the huge gap in rice breeding endeavour required in response to global rise in salinity.

## Supplementary information

**Additional file 1: Table S1.** QTLs/genes for salt-tolerance on all chromosome in rice

## Data Availability

Not applicable.
