# Supplementary material for: Harmonizing technological advances in phenomics and genomics for enhanced salt tolerance in rice from a practical perspective
Source: Rice (N Y). 2019 Dec 4;12:89. doi: 10.1186/s12284-019-0347-1 (PMC6892996; doi:10.1186/s12284-019-0347-1)
Supplement: Supplementary file 1 — Additional file 1: Table S1. QTLs/genes for salt-tolerance on all chromosome in rice [file 12284_2019_347_MOESM1_ESM.docx]

**Table S1. QTLs/genes for salt-tolerance on all chromosome in rice**

| **Chromosome No.** | **Gene/QTLs** | **Trait** | **Phenotypic**  **Variation (%)** | **Flanking/**  **nearest Marker** | **References** |
| --- | --- | --- | --- | --- | --- |
| 1 | *SAL T* | Stress induced protein | - | RG146B | Claes *et al.* (1990) |
|  | *SALTOL* | K absorption | 80.2 | P3/M9-8-*SALTOL* | Gregorio (1997) |
|  | *SALTOL* | Na absorption | 64.6 | P3/M9-8-*SALTOL* |  |
|  | *SALTOL* | Na/K ratio | 64.3 | P3/M9-8-*SALTOL* |  |
|  | *Q _Na_* | High sodium uptake |  |  | Flowers *et al.* (2000) |
|  | Trait based QTL | Shoot Na/K ratio | 9.14 | C86-RM212 | Lang *et al.* (2001 b) |
|  | Trait based QTL | Na uptake | 8.9 | E12M55-3 | Koyama *et al.* (2001) |
|  | Trait based QTL | Kconcentration | 10.6 | E12M37-1 |  |
|  | Trait based QTL | Na/K ratio | 9.1 | E12M57-1 |  |
|  | *SALTOL* | Na uptake | 39.2 | RM140-C1733S | Bonilla *et al.* (2002) |
|  | *SALTOL* | K uptake | 43.9 | RM140-C1733S |  |
|  | *SALTOL* | Na/K ratio | 43.2 | RM140-C1733S |  |
|  | *SALTOL* | Ion uptake and Na/K ratio | 0.44 | RM 140 | Niones (2004) |
|  | *SALTOL* | Ion uptake and Na/K ratio | 0.44 | CP03970-CP3224 |  |
|  | *SalTol*1-1 | Seedling Stage | 12.5 | RM8094-RM493 | Islam *et al.* (2011) |
|  | *qCl^-^ SV1-1* | Cl in stem at vegetative stage | 55.72 | RM572-RM294 | Ammar (2004) |
|  | *qNa^+^LR8-1* | Na in leaf at vegetative stage | 55.18 | RM3395-RM281 |  |
|  | *qNa^+^LV3-1* | Na in leaf at vegetative stage | 53.8 | RM563-RM186 |  |
|  | *qNa^+^SV2-1* | Na in stem at vegetative stage | 49 | RM145-RM5699 |  |
|  | *qSDS-1* | Seedling survival | 18 | C813-C86 | Lin *et al.* (2004). |
|  | *qSKC-1* | Shoot K | 40.1 | C1211-S2139 |  |
|  | *qRNTQ-1* | Root Na | 12.4 | C813-C86 |  |
|  | *qSKC-1* | Shoot K |  | K159-K061 | Ren *et al.* (2005) |
|  | *qST1* |  | 27.76 | Est1-2 and RZ569A | Lee *et al.* (2006) |
|  | *QKr1.1* | Potassium in Root | 17 | RM200-RM220 | Ahmadi *et al.* (2011) |
|  | *QKr1.2* |  | 30 | RM473A-RM128 |  |
|  | *QNas1* | Sodium in Root | 15 | RM128-RM212 |  |
|  | *QNas/Ks1* | Na^+^/K^+^ ratio in shoot | 19 | RM23-RM5 |  |
|  | *QNar/Kr1* |  | 18.4 | RM473A-RM128 |  |
|  | *qSKC1* | Shoot K^+^ concentration | 14.3 | RM7341-RM7419 | Wang *et al.* (2012) |
|  | *qRDW-1* | Root dry weight | 0.26 | RM1268-RM8231 | Ghomi *et al.* (2013) |
|  | *qSTR-1* | Standard tolerance ranking | 12.41 | RM8132-E36-M60-7 |  |
|  | *qSKC-1* | Shoot K concentration | 16.69 | E38-M59-4-RM1287 |  |
|  | *qRL1* | Chlorophyll content | 13.63 | RM 8068-RM 8231 | Sabouri and Sabouri (2008) |
|  | *qFWSH-1* | Fresh weight shoot | 22.44 | RM8235-RM8144 |  |
|  | *qNAUP-1a* | Na^+^ uptake | 13.03 | RM562-RM543 |  |
|  | *qNAUP-1b* | Na^+^ uptake | 22.17 | RM8068-RM8231 |  |
|  | *qBI-1a* | Biomass |  |  | Sabouri and Sabouri (2008) |
|  | *qBI-1b* | Biomass |  |  |  |
|  | *QTL1* | Seedling salt tolerance | 5.77 | RM84-RM259 | Ammar *et al.* (2007) |
|  | *Qsf1.4* | Spikelet Fertility |  | RM576-RM312 | Chai *et al.* (2013) |
|  | *Qgw1.4* | Grain Weight |  | RM576-RM579 |  |
|  | *Qgyp1.4* | Grain Yield per plant |  | RM576-RM579 |  |
|  | *qPH1.1s* | Plant height | 17.5 | RM212 | Mohammadi *et al.* (2013) |
|  | *qPL1.1s* | Panicle Length | 6.2 | RM212 |  |
|  | *QK1* |  |  |  | Flowers *et al.* (2000) |
|  | *qPHl1.1* | Plant height | 48.7 | RM128-RM472 | Hossain et al (2015) |
|  | *qPH1.1* | Plant height | 47.1 | RM128-RM472 |  |
|  | *qPF1.4* | Pollen fertility | 13.4 | RM246-RM6648 |  |
|  | *qPF1.5* | Pollen fertility | 13.7 | RM128-RM472 |  |
|  | *qNa1.7* | Na/K ratio | 13.5 | RM1349-RM7250 |  |
|  | *qNaKR1.8* | Na/K ratio | 11.0 | RM237-RM7250 |  |
|  | *qSFW-1a-CK* | Shoot fresh weight | 13.24 | RM323-RM8144 | La *et al.* (2014) |
|  | *qSFW-1b-CK* | Shoot fresh weight | 25.13 | RM449-RM8094 |  |
|  | *qRK-1-CK* | Root K^+^ | 5.52 | RM1287-RM8094 |  |
|  | *qSN-1-CK* | Shoot Na^+^ | 10.67 | RM3412-RM220 |  |

| 2 | Trait based QTL | Shoot Na/K ratio | 5.25 | C747 | Lang *et al.* (2001 b) |
| --- | --- | --- | --- | --- | --- |
|  | Trait based QTL | Shoot Na concentration | 7.22 | RM240-RM213 |  |
|  | *Qk^+^SV9-1* | Stem K at vegetative stage | 7.51 | RM41-RM219 | Ammar (2004) |
|  | *qNa^+^SV8-1* | Stem Na at vegetative stage | 53.63 | RM3395-RM281 |  |
|  | *qCl^-^SV2-1* | Stem Cl at vegetative stage | 42.5 | RM145-RM5699 |  |
|  | Trait based QTL | Tiller number | 12 | C747-R3393 | Takehisa *et al.* (2004) |
|  | Trait based QTL | Tiller number | 12-23 | C1408-C560 |  |
|  | Qsdw-2 | Shoot dry weight | 17.88 | RM279-RM5911 | Ghomi *et al.* (2013) |
|  | Qskc-2 | Shoot K concentration | 1.95 | RM262-RM7624 |  |
|  | Qpl-2 | Plant stand | 16.45 | RM5699-RM262 | Sabouri *et al.* (2008) |
|  | qNA-2a | Na^+^ Content | 12.59 | RM8264-RM262 | Sabouri *et al.* (2009) |
|  | qNA-2b |  | 12.57 | RM7426-RM236 |  |
|  | Qsf2.5 | Spikelet Fertility |  | RM71-RM424 | Chai *et al.* (2013) |
|  | Qsf2.11 | Spikelet Fertility |  | RM6-RM208 |  |
|  | Qgw2.11 | Grain Weight |  | RM6-RM208 |  |
|  | Qgyp2.5 | Grain Yield per plant |  | RM71-RM424 |  |
|  | Qgyp2.11 | Grain Yield per plant |  | RM6-RM208 |  |
|  | qNAK-2 | Na^+^/K^+^ ratio |  |  | Ming-zhe *et al.*  (2005) |
|  | QK2 |  |  |  | Flowers *et al.*  (2000) |
|  | qPL2.1s | Panicle length | 8.3 | RM324 | Mohammadi *et al.* (2013) |
|  | qFRSP2.1s | Number of fertile spikelets | 5.7 | RM555 |  |
|  | qGY2.1s | Grain yield per plant | 3.6 | RM174 |  |
|  | qSPFR2.1s | Spikelet fertility | 7.9 | RM555 |  |
| 3 | Trait based QTL | Na absorption | 17.1 | P1/M5-3-P3/M9-1 | Gregorio (1997) |
|  | Trait based QTL | Na absorption | 16 | P1/M10-6-P1/M7-10 | Gregorio (1997) |
|  | Trait based QTL | Root weight | 9.34 | R3156-C563 | Lang *et al.* (2001 b) |
|  | *qNa^+^/K^+^LR3-1* | Leaf Na/K at reproductive stage | 52.63 | RM563-RM186 | Ammar (2004) |
|  | *qNa^+^/K^+^LR8-1* | Leaf Na/K at reproductive stage | 51.03 | RM3395-RM281 |  |
|  | Trait based QTL | Shoot length | 14-24 | C944-C595 | Takehisa *et al.* (2004) |
|  | Trait based QTL | Shoot length | 14-17 | R250-C136 |  |
|  | *Qst-3* |  | 9.16 | RG179-RZ596 | Lee *et al.* (2006) |
|  | *Qlb-3* | Leaf bronzing | 83 | R1925 | Takehisa *et al.* (2006) |
|  | *QKr3* | Potassium in Root | 14 | RM251-RM282 | Ahmadi *et al.* (2011) |
|  | *Qsnc3* | Shoot Na concentration | 13.1 | RM7370-RM6832 | Wang *et al.* (2012) |
|  | *Qsfw-3* | Shoot fresh weight | 0.02 | RM60-RM489 | Ghomi *et al.* (2013) |
|  | *Qsdw-3* | Shoot dry weight | 0.72 | RM60-RM489 |  |
|  | *Qbm-3* | Biomass | 3.9 | RM60-RM489 |  |
|  | *Qchlc-3* | Chlorophyll content | 14.50 | RM1022-RM6283 | Sabouri *et al.* (2008) |
|  | *Qshl-3* | Shoot Length | 23.57 | RM7389-RM7000 |  |
|  | *Qfwsh-3* | Fresh Weight Shoot | 22.97 | RM1022-RM6283 |  |
|  | *Qfwro-3a* | Fresh Weight root | 20.91 | RM1022-RM6283 |  |
|  | *Qfwro-3b* | Fresh Weight root | 23.21 | RM6283-RM6832 |  |
|  | *Qdwsh-3* | Dry weight shoot | 23.21 | RM1022-RM6283 |  |
|  | *Qdwro-3* | Dry weight root | 21.41 | RM1022-RM6283 |  |
|  | *qNaUP-3* | Na^+^ uptake | 13.62 | RM416-RM5626 |  |
|  | *Qkup-3* | K^+^ uptake | 22.15 | RM1022-RM6283 |  |
|  | *qNaKUP-3* | Na^+^/K^+^ uptake ratio | 9.03 | RM6832-RM7389 |  |
|  | *qDM-3* | Dry Mass | 20.50 | RM1022-RM6283 | Sabouri *et al.*  (2009) |
|  | *qSTR-3a* | Standard tolerance ranking | 13.44 | RM1022-RM6283 |  |
|  | *qSTR-3b* |  | 24.51 | RM6832-RM7389 |  |
|  | *qNA-3* | Na^+^ Content | 16.34 | RM6832-RM7389 |  |
|  | *qNAK-3* | Na^+^/K^+^ ratio | 18.52 | RM6832-RM7389 |  |
|  | *QTL43* | Seedling salt tolerance | 25.8 | RM563-RM186 | Ammar *et al.* (2007) |
|  | *qSDW-3-CK* | Shoot dry weight | 14.56 | *RM2593-RM563* | La *et al.* (2014) |
|  | qPH3.1s | Plant height | 12 | RM520 | Mohammadi *et al.* (2013) |
|  | qPL3.1s | Panicle length | 8.5 | RM130 |  |
|  | qSTSP3.1s | Number of sterile spikelets | 8 | RM282 |  |
| 4 | Trait based QTL | K absorption | 83.5 | RG375-P4/M3-2 | Gregorio (1997) |
|  | *Q _Na/K_* | Na/K discrimination |  |  | Flowers *et al.* (2000) |
|  | Trait based QTL | Kuptake | 6.8 | E12M65-1 | Koyama *et al.* (2001) |
|  | Trait based QTL | K concentration | 8.8 | E15M53-2 |  |
|  | Trait based QTL | Na concentration | 6.7 | E12M73-1; E12M75-5;  E15M50-5; E12M79-1; |  |
|  | Trait based QTL | Na/K ratio | 9.6 | E12M65-1 |  |
|  | *qNa^+^LR8-1* | Leaf Na at reproductive stage | 47.59 | RM3395-RM281 | Ammar (2004) |
|  | *qNa^+^SV1-1* | Stem Na at reproductive stage | 52.27 | RM572-RM294 |  |
|  | *qNa^+^/K^+^SV-1* | Stem Na/K at vegetative stage | 46.57 | RM3732-RM145 |  |
|  | *qNa^+^/K^+^SV-2* | Stem Na/K at vegetative stage | 45.5 | RM3732-RM145 |  |
|  | *qNa^+^/K^+^SV-3* | Stem Na/K at vegetative stage | 42.88 | RM145-RM5699 |  |
|  | *Qrkc-4* | Root K concentration | 21.6 | C891-C513 | Lin *et al.* (2004) |
|  | *QKs4* | Potassium in Shoot | 19 | RM261d-RM273 | Ahmadi *et al.* (2011) |
|  | *QKr4* | Potassium in Root | 9 | RM241-RM348 |  |
|  | *Qnar/Kr4* | Na^+^/K^+^ ratio in shoot | 9 | RM241-RM348 |  |
|  | *Qskc4* | Shoot K^+^ concentration | 18.9 | RM5687-RM273 | Wang *et al.* (2012) |
|  | *Qrkc4* | Root K^+^ concentration | 8.5 | RM518-RM16535 |  |
|  | *Qrfw-4a* | Root fresh weight | 7.81 | RM8213-E36-M59-5 | Ghomi *et al.* (2013) |
|  | *Qrfw-4b* | Root fresh weight | 19.06 | E36-M59-5-E37-M60-3 |  |
|  | *Qrdw-4* | Root dry weight | 5.03 | E36-M60-10-E38-M61-4 |  |
|  | *Qstr-4* | Standard tolerance ranking | 5.48 | E37-M61-9-RM1359 |  |
|  | *Qrl-4* | Chlorophyll Content | 11.56 | RM5473-RM551 | Sabouri *et al.* (2008) |
|  | *QTL3* | Seedling salt tolerance | 5.13 | RM5320-RM3648 | Ammar *et al.* 2007 |
|  | *QTL4* | Seedling salt tolerance | 7.11 | RM3648-RM280 |  |
|  | *Qgw4.7* | Grain Weight |  | RM303-RM280 | Chai *et al.* (2013) |
|  | qDTF4.1s | Days to flowering | 5.9 | RM252 | Mohammadi *et al.* (2013) |
|  | qPN4.1s | Number of panicles | 5.1 | RM518 |  |
|  | qSTW4.1s | Straw dry weight (g) | 7.4 | RM335 |  |
|  | qFRSP4.1s | Number of fertile spikelets | 6 | RM335 |  |
|  | qTSP4.1s | Total spikelets number | 10.4 | RM335 |  |
|  | qGY4.1s | Grain yield per plant | 6 | RM335 |  |
|  | qSTR-4-CK | Standard tolerance ranking | 39 | RM1359-RM127 4 | La *et al.* (2014) |
| 5 | *Qsdm-5* | Seedling dry matter | 17.9 | RZ70-RZ225 | Prasad *et al.* (2000) |
|  | *QKs5* | Potassium in Shoot | 22 | RM413-RM289 | Ahmadi *et al.* (2011) |
|  | *Qnar/Kr5* | Na^+^/K^+^ ratio in root | 27.6 | RM122-RM413 |  |
|  | *Qsfw5a* | Shoot fresh weight | 9.91 | E36-M59-10-RM440 | Ghomi *et al.* (2013) |
|  | *Qsfw5b* | Shoot fresh weight | 22.89 | RM459-RM3800 |  |
|  | *Qbm-5a* | Biomass | 16.27 | E36-M59-10-RM440 |  |
|  | *Qbm-5b* | Biomass | 15.3 | RM459-RM3800 |  |
|  | *Qshl-5* | Shoot Length | 19.57 | RM13-RM164 |  |
|  | *Qsnk-5* | Shoot Na/K ratio | 0.32 | RM6320-E38-M61-11 |  |
|  | *Qrl-5* | Chlorophyll Content | 14.80 | RM421-RM480 | Sabouri *et al.* (2008) |
|  | *Qdwro-3* | Dry weight root | 21.41 | RM1022-RM6283 |  |
|  | *Qdwro-5a* | Dry weight root | 21.74 | RM421-RM480 |  |
|  | *Qdwro-5b* | Dry weight root | 22.55 | RM480-RM440 |  |
|  | *qK-5a* | K^+^ Content | 17.16 | RM421-RM480 | Sabouri *et al.* (2009) |
|  | *qK-5b* |  | 12.35 | RM480-RM440 |  |
|  | *QTL44* | Seedling salt tolerance | 8.7 | RM233B-RM334 | Ammar *et al.* (2007) |
|  | *qGW5.4* | Grain Weight |  | RM405-RM163 | Chai *et al.* (2013) |
|  | *qGYP5.4* | Grain Yield per plant |  | RM405-RM163 |  |
|  | qPH5.1s | Plant height | 5.2 | RM538 | Mohammadi *et al.* (2013) |
|  | qSPFR2.5s | Spikelet fertility | 6 | RM161 |  |
|  | qTGW5.1s | 1000-grain weight | 4.6 | RM413 |  |
| *6* | *qSGEM-6* | Seed germination | 16.3 | RZ398-RG213 | Prasad *et al.* (2000) |
|  | *qSRTL-6* | Seedling root length | 18.9 | RG 162-RG 653 |  |
|  | *qSDM-6* | Seedling dry matter | 16.7 | CDO544-Amy2A |  |
|  | *qSV-6* | Seedling vigour | 15.8 | CDO544-Amy2A |  |
|  | *Q_K1_* | K uptake |  |  | Flowers *et al.* (2000) |
|  | Trait based QTL | Dry mass | 9.7 | E12M55-2 | Koyama *et al.* (2001) |
|  | Trait based QTL | Kuptake | 7.6 | OSR19;E12M80-2 |  |
|  | Trait based QTL | Na concentration | 6.4 | E12M35-2 |  |
|  | *qSDS-6* | Seedling survival | 17 | C214-R2549 | Lin *et al.* (2004) |
|  | *QNas6* | Sodium in Root | 24 | RM3-RM528 | Ahmadi *et al.* (2011) |
|  | *qRKC6.1* | Root K^+^ concentration | 2.7 to 3.6 | RM340-RM6811 | Wang *et al.* (2012) |
|  | *qRKC6.2* |  |  | RM6811-RM176 |  |
|  | *qSFW-6* | Shoot fresh weight | 6.51 | RM340-E36-M61-3 | Ghomi *et al.* (2013) |
|  | *qBM-6* | Biomass | 6.0 | E36-M60-6-RM5371 |  |
|  | *qSHL-6* | Shoot length | 14.63 | RM402-RM549 |  |
|  | *qNAKUP-6* | Na^+^/K^+^ uptake ratio | 12.35 | RM3827-RM340 | Sabouri and Sabouri (2008) |
|  | *qSTR-6* | Standard tolerance ranking | 17.51 | RM3727-RM340 | Sabouri *et al.*  (2009) & Ming-zhe *et al.*  (2005) |
|  | *qK-6* | K^+^ content | 14.46 | RM3827-RM340 |  |
|  | *qNA-6* | Na^+^ Content | 11.92 | RM3827-RM5371 |  |
|  | *qNAK-6* | Na^+^/K^+^ ratio | 16.68 | RM3827-RM340 |  |
|  | *qGW6.2* | Grain Yield per plant |  | RM253-RM527 | Chai *et al.* (2013) |
|  | *qGYP6.5* | Grain Yield per plant |  | RM541-RM528 |  |
|  | *qGYP6.2* | Grain Yield per plant |  | RM253-RM527 |  |
|  | *qSNK-6-CK* | Shoot Na/K ratio | 8.0 | *RM141-RM253* | La *et al.* (2014) |
|  | qDTF6.1s | Days to flowering | 10.6 | RM136 | Mohammadi *et al.* (2013) |
|  | qPN6.1s | Number of panicles | 6.8 | RM275 |  |
|  | qFRSP6.1s | Number of fertile spikelets | 6 | RM275 |  |
|  | qGY6.1s | Grain yield per plant | 6.7 | RM275 |  |
|  | qTGW6.1s | 1000-grain weight | 3.9 | RM275 |  |
| 7 | Salt tolerance | Score based salt-tolerance | - | RG4 | Zhang *et al.* (1995) |
|  | Trait based QTL | 1000 grain weight | - | RG4 |  |
|  | *qSGEM-7* | Seed germination | 19.5 | CDO59-RG477 | Prasad *et al.* (2000) |
|  | Trait based QTL | Shoot Na/K ratio | 5.86 | RM214-R1789 | Lang *et al.* (2001 b) |
|  | Trait based QTL | Shoot length | 16-22 | R2401-R1488 | Takehisa *et al.* (2004) |
|  | Trait based QTL | Shoot length | 23 | C1057-R565 |  |
|  | *qSDS-7* | Seedling survival | 13.9 | R2401-L538T7 | Lin *et al.* (2004) |
|  | *qSNC-7* | Shoot Na concentration | 48.5 | C1057-R2401 |  |
|  | *qSNTQ-7* | Shoot Na total quantity | 16.1 | C1057-R2401 |  |
|  | *qRKC-7* | Root K concentration | 17.8 | C1057-R2401 |  |
|  | *qRKTQ-7* | Root K total quantity | 17.3 | C1057-R2401 |  |
|  | *qSTR7* | Salt tolerance rating | 8.6 | RM11-RM5380 | Wang *et al.* (2012) |
|  | *qRDW-7* | Root dry weight | 14.6 | E38-M61-6-E37-M61-12 | Ghomi *et al.* (2013) |
|  | *qRL-7* | Root Length | 16.21 | RM1048-RM11 | Sabouri and Sabouri (2008) |
|  | *qDWSH-7* | Dry Weight Shoot | 23.17 | RM5481-RM11 |  |
|  | *qGYP7.3* | Grain Yield per plant |  | RM501-RM418 | Chai *et al.* (2013) |
|  | *qSDW-7-CK* | Shoot dry weight | 17.88 | *RM5481-RM5720* | La *et al.* (2014) |
|  | qPH7.1s | Plant height | 11.8 | RM336 | Mohammadi *et al.* (2013) |
|  | qSTW7.1s | Straw dry weight | 7.2 | RM432 |  |
|  | qSTSP7.1s | Number of sterile spikelets | 5.8 | RM11 |  |
|  | qTSP7.1s | Total spikelets number | 6.9 | RM432 |  |
|  | *qPH7.1* | Plant height | 22.5 | RM51-RM1243 | Hossain et al (2015) |
|  | *qPL7.4* | Panicle length | 30.0 | RM180-RM3635 |  |
|  | *qPL7.4* | Panicle length | 35.1 | RM180-RM3635 |  |
|  | *qTNl7.2* | Tiller number | 16.3 | RM51-RM1243 |  |
|  | *qTNl7.3* | Tiller number | 12.0 | RM180-RM7110 |  |
| 8 | Trait based QTL | Salt tolerance at vegetative  and reproductive stage | - | RM223 | Lang *et al.* (2001 a) |
|  | *qCl^-^LR2-1* | Leaf Cl at reproductive stage | 26.26 | RM145-RM5699 | Ammar (2004) |
|  | *qCl ^-^LV3-1* | Leaf Cl at vegetative stage | 48.51 | RM563-RM186 |  |
|  | *qNa^+^/K^+^ SV 8-1* | Stem Na/K at vegetative stage | 51.78 | RM3395-RM281 |  |
|  | *qNa^+^/K^+^SV3-1* | Stem Na/K at vegetative stage | 47.51 | RM563-RM186 |  |
|  | *QKr8* | Potassium in Root | 20 | RM149-RM264 | Ahmadi *et al.* (2011) |
|  | *QNar/Kr8* | Na^+^/K^+^ ratio in root | 16.7 | RM149-RM264 |  |
|  | *qCHL-8* | Chlorophyll | 15.78 | E37-M61-7-RM152 | Ghomi *et al.* (2013) |
|  | *qSTR-8* | Standard tolerance ranking | 19.66 | RM7027-RM8264 |  |
|  | *qSNK-8* | Shoot Na/K ratio | 15.12 | RM3572-RM404 |  |
|  | *qKUP-8* | K^+^ uptake | 38.22 | RM4955-RM152 | Sabouri and Sabouri (2008) |
|  | *qDM-8* | Dry Mass | 20.24 | RM4955-RM152 | Sabouri *et al.*(2009) |
|  | *qDWS-8* | Dry weight of shoot | 7.5 | RM223–RM152 | Ming-zhe *et al.* (2005) |
|  | *SalTol*8-1 | Seedling Stage | 29.0 | RM25-RM210 | Islam *et al.* (2011) |
|  | qSTW8.1s | Straw dry weight | 4.1 | RM25 | Mohammadi *et al.* (2013) |
|  | qGY8.1s | Grain yield per plant | 7.4 | RM477 |  |
|  | qTGW8.1s | 1000-grain weight | 25.5 | RM477 |  |
|  | *qTN8.1* | Tiller number | 14.4 | RM6369-RM547 | Hossain et al (2015) |
|  | *qTN8.1* | Tiller number | 11.3 | RM3115-RM44 |  |
|  | *qBM8.2* | Biomass | 17.9 | RM3215-RM44 |  |

| 9 | Q_k2_ | K^+^ uptake |  |  | Flowers *et al.* (2000) |
| --- | --- | --- | --- | --- | --- |
|  | Trait based QTL | Root weight | 6.35 | C397-C1454 | Lang *et al.* (2001 b) |
|  | Trait based QTL | Kuptake | 19.6 | E12M55-4 | Koyama *et al.* (2001) |
|  | *qRNC-9* | Root Na concentration | 16.7 | R1751-R2638 | Lin *et al.* (2004) |
|  | *qSKC9* | Shoot K+ concentration | 9.2 | RM5688-RM444 | Wang *et al.* (2012) |
|  | *qSTR-9* | Standard tolerance ranking | 21.7 | E36-M61-2-RM257 | Ghomi *et al.* (2013) |
|  | *qRL-9* | Root length | 15.59 | RM219-RM7038 |  |
|  | *qSHL-9* | Shoot length | 9.99 | E37-M60-13-E36-M60-1 |  |
|  | *qSNC-9* | Shoot Na concentration | 2.14 | RM201-RM215 |  |
|  | *qSNK-9* | Shoot Na/K ratio | 9.88 | RM288-RM278 |  |
|  | *qRL-9a* | Root Length | 14.12 | RM1553-RM5702 | Sabouri and Sabouri (2008) |
|  | *qRL-9b* | Root Length | 11.51 | RM7424-RM5702 |  |
|  | *qDWRO-9a* | Dry weight root | 27.43 | RM1553-RM7424 |  |
|  | *qDWRO-9b* | Dry weight root | 25.50 | RM7424-RM5702 |  |
|  | *qNAUP-9a* | Na^+^ Uptake | 17.71 | RM1553-RM7424 |  |
|  | *qNAUP-9b* | Na^+^ Uptake | 16.95 | RM7424-RM5702 |  |
|  | *qDWS-9* | Dry weight of shoot | 11.5 | RM278–RM215 | Ming-zhe *et al.* (2005) |
|  | *qSNK-9-CK* | Shoot Na/K ratio | 23.5 | RM242-RM460 | La *et al.* (2014) |
|  | *qRNK-9-CK* | Root Na/K ratio | 9.33 | RM105-RM287 |  |
|  | qPN9.1s | Number of panicles | 5.8 | RM242 | Mohammadi *et al.* (2013) |
|  | qSTW9.1s | Straw dry weight | 4.2 | RM242 |  |
|  | qTSP9.1s | Total spikelets number | 5.1 | RM410 |  |
| 10 | Trait based QTL | Na absorption | 35.6 | P1/M3-10-P1/M3-8 | Gregorio (1997) |
|  | Trait based QTL | Na/K ratio | 86.1 | G291-P1/M7-8 |  |
|  | *qSDM-10* | Seedling dry matter | 13.5 | RZ625-RZ500 | Prasad *et al.* (2000) |
|  | *qRKC10* | Root K+ concentration | 9.1 | RM3773-RM8202 | Wang *et al.* (2012) |
|  | *qSHL-10* | Shoot length | 4.13 | RM2863-E36-M61-13 | Ghomi *et al.* (2013) |
|  | *qSHL-10* | Shoot Length | 19.19 | RM7545-RM4455 | Sabouri and Sabouri (2008) |
|  | *qNAUP-10* | Na+ uptake | 13.84 | RM7545-RM4455 |  |
|  | *qSNP10.6* | Spikelet number per panicle |  | RM311-RM294a | Chai *et al.* (2013) |
|  | *SalTol*10-1 | Seedling Stage | 20.2 | RM25092-RM25519 | Islam *et al.* (2011) |
|  | qDTF10.1s | Days to flowering | 14 | RM258 | Mohammadi *et al.* (2013) |
|  | qFRSP10.1s | Number of fertile spikelets | 5.3 | RM496 |  |
|  | qSPFR2.10s | Spikelet fertility | 6.7 | RM258 |  |
|  | qTGW10.1s | 1000-grain weight | 16.2 | RM258 |  |
| 11 | Trait based QTL | Shoot weight | 14.38 | RM209-RM206 | Lang *et al.*  (2001 b) |
|  | *qLB-11* | Leaf bronzing | 8 | C1350-C477 | Takehisa *et al.* (2006) |
|  | *qSNC11* | Shoot Na^+^ concentration | 16.1 | RM286-RM6288 | Wang *et al.* (2012) |
|  | *qSF11.5* | Spikelet Fertility |  | RM21-RM206 | Chai *et al.* (2013) |
|  | *qSF11.6* | Spikelet Fertility |  | RM209-RM254 |  |
|  | *qGYP11.7* | Grain Yield per plant |  | RM206-RM224 |  |
| 12 | Trait based QTL | K absorption | 21.2 | P1/M1-3-P2/M1-3 | Gregorio (1997) |
|  | Trait based QTL | Na/K ratio | 18.5 | P1/M1-3-P2/M1-3 |  |
|  | Trait based QTL | Shoot K concentration | 17.45 | G24-R1684 | Lang *et al.* (2001 b) |
|  | Trait based QTL | Shoot Na/K ratio | 8.81 | G24-R1684 |  |
|  | qSTR-12 | Standard Tolerance Ranking | 2.14 | RM2935-E36-M60-4 | Ghomi *et al.* (2013) |
